# Medical College Fees

**Published:** 1869-07-15

**Authors:** 


### *Rush Medical College.*

The Annual Catalogue and announcement of the course for 1869 and 1870 will be issued soon. Correspondents please notice.

### *Medical College Fees.*

The JOURNAL has not space for its multitudinous communications on this subject, nor the editors time for private reply to the numerous circulars and letters calling their attention to the subject, and announcing the views of colleges, societies and individuals. We have abundant confidence in the business capacity of the Trustees of Rush Medical College, and we opine their doctrine is that "Entangling alliances should be avoided." The interests of the College and the profession are identical. The idea of the Faculty we know is to furnish the best possible instruction, both didactic and clinical, irrespective of fees, whether high or low. It is a notable fact that the chairs of Medical Colleges are filled (when *filled* at all) by men who do not seek or occupy them from pecuniary motives.

In cities of any considerable size, the contingent advantages of these positions often induce gentlemen to occupy them even at some direct pecuniary sacrifice. In small cities, where these contingent advantages are not presented, the Faculties, unless salaried, are driven to seek direct emolument from advanced fees and diminished expenditure. Otherwise, as is too often the case, the chairs are sought and secured by little men whose ambition is to hold the title of Professor, as the only chance for notoriety. The next step with these men is to get into some general convention, and then clamor about "the elevation of the profession.'' They seek the power of what should be purely scientific associations to convert their impecunious titles into money-producing realities. Thus they attempt (and not infrequently succeed) to convert the scientific association into a trades-union, with pains and penalties.

"Let every tub stand on its own bottom." If every little city and hamlet which holds a half-dozen superfluous physicians chooses to incorporate them into a Medical Faculty, let it so be done, but let the incorporate then understand that they must depend upon themselves for subsequent success, and not upon loose and flatulent resolutions adopted by this or that organization serenely oblivious of the eleventh commandment: "Mind your own business." We wish somebody would explain to us why it is that after a longer or shorter interval, spent by a certain class of medical men in clamoring about the imperfections of medical teaching, and filling all ears with their jeremiads, objurgations and *twaddle*, the next thing we know of them they start a medical college where it is less called for than the proverbial "fifth wheel of a wagon," or "the little hole for the kitten, where there is already a big hole for the cat."

No wonder the professional trades-unions groan in spirit, and the apostolic "elevators" are disquieted thereby. It pleases us to see that the subject is temporarily postponed, by general consent. May it sleep well!

